# MEMA: Multimodal Aesthetic Evaluation of Music in Visual Contexts

**DOI:** 10.3390/s26041395

**Published:** 2026-02-23

**Authors:** Huaye Zhang, Chenglizhao Chen, Mengke Song, Tingting Chen, Diqiong Jiang, Lichun Liu, Xinyu Liu

**Affiliations:** Qingdao Institute of Software, College of Computer Science and Technology, China University of Petroleum (East China), Qingdao 266580, China; 2306030225@s.upc.edu.cn (H.Z.); djiang@upc.edu.cn (D.J.);

**Keywords:** multimodal learning, aesthetic quality assessment, audio-visual alignment, cross-attention, video soundtrack evaluation

## Abstract

Recent technologies such as music retrieval, soundtrack generation, and video understanding have developed rapidly. Consequently, the aesthetic evaluation of video soundtracks has become an important research topic in academia. Soundtracks are key elements in shaping the emotional atmosphere and driving the narrative rhythm. Therefore, they require systematic methods to assess their artistic coordination with visual content. However, existing approaches mostly focus on evaluating the quality of the music itself. They often lack the ability to model the deeper aesthetic synergy between audio and visuals. To address this gap, we propose MEMA, a new soundtrack aesthetic evaluation model. MEMA employs a two-stage training strategy. The first stage builds a crossmodal imagination mechanism using a Conditional Variational Autoencoder. This method achieves bidirectional semantic reconstruction between audio and visuals. The second stage introduces a Guided Cross-Attention Alignment Module. This module enhances the model’s focus on key narrative moments in video. To facilitate this research, we also construct VMAE-Sets. It is the first large-scale dataset dedicated to soundtrack aesthetic evaluation. Finally, MEMA performs scoring and textual evaluation along three core aesthetic dimensions. Experimental results demonstrate that MEMA outperforms existing methods, achieving average improvements of 18.137% in LCC and 17.866% in SRCC compared to the strongest baseline. These findings confirm its superior audio–visual narrative alignment, demonstrating high consistency with human judgments.

## 1. Introduction

The rapid development of multimodal technologies, including video generation [1,2,3,4,5,6,7] and video understanding [8,9,10,11,12,13,14], has resulted in automated audio–visual content creation. Large-scale vision language models such as CLIP [15] have demonstrated the effectiveness of crossmodal alignment through contrastive learning. This progress introduces a critical challenge beyond simple generation: evaluating the artistic coordination between soundtracks and visuals. This task is a form of aesthetic assessment, which judges subjective qualities like emotional resonance and creative originality. Within computer vision and multimedia, aesthetic assessment ensures that machine-generated content aligns with human perceptions of beauty, spanning domains like image and video aesthetic assessment. Consequently, a clear need has emerged for systematic methods and high-quality datasets to specifically evaluate video soundtrack aesthetics. However, aesthetic evaluation of the audio–visual relationship remains a critical challenge.

At the methodological level, current evaluation approaches exhibit significant limitations. Traditional music quality assessment methods rely solely on audio signals. They treat audio signals in isolation and focus solely on physical or perceptual metrics such as the signal-to-noise ratio, loudness, and rhythm stability [16,17,18]. These approaches overlook the functional role of music in cinematic contexts. Music serves as a narrative device that works together with visual elements to construct emotional tension and rhythmic structure. As a result, such methods cannot capture the expressive intent and narrative value of soundtracks in specific scenes. Although recent multimodal evaluation methods attempt to integrate video and audio information, most remain limited to low-level feature fusion. A common approach is to extract features from image frames and audio segments. These features are then concatenated directly or processed by shallow attention mechanisms for joint modeling. While these techniques offer modest improvements in capturing inter-modal correlations, they fall short in modeling the dynamic interplay between musical progression and narrative development. They also fail to capture the aesthetic coupling between timbre, rhythm, and visual atmosphere [19].

On the data side, there is currently no high-quality aesthetic evaluation dataset that is comprehensive, fine-grained, and focused on artistic expression. Compared to general video–music alignment tasks, film soundtracks place greater emphasis on high-level emotional alignment between the music and the visual narrative. This alignment goes beyond crossmodal information matching. It involves the synergy between musical atmosphere and visual storytelling across emotional rhythms, narrative structures, and thematic metaphors. However, existing datasets are insufficient to support in-depth aesthetic evaluations between video and music. For example, SymMV [4] provides annotations for video–music alignment but primarily contains narrow-range samples using a solo piano. This limitation restricts its musical diversity. BGM909 [4] offers high-quality audio–video–text triplets but focuses mainly on semantic-level alignment. It lacks descriptions of whether the soundtrack emotionally resonates with the visuals. Similarly, HarmonySet [20] is designed for aesthetic evaluation tasks but mainly covers short-video scenarios. It fails to capture the more nuanced and complex aesthetic requirements of film scoring. At the core of this problem lies a significant bottleneck in obtaining high-quality aesthetic annotations. Expert annotation requires interdisciplinary knowledge of both music composition and film theory. This makes large-scale dataset construction costly and time-consuming. As a result, existing datasets often only provide coarse-grained labels and fail to distinguish between different aesthetic dimensions.

To address the above challenges, we propose a solution that includes both a new evaluation model and a dataset. For the model, we introduce MEMA (Multimodal Aesthetic Evaluation of Music), which captures the artistic relationships between audio and visuals through crossmodal imagination. The model employs a Conditional Variational Autoencoder (CVAE) [21] for bidirectional audio–visual reconstruction. This mechanism enables the model to learn deep features by imagining one modality from another. MEMA decomposes aesthetic judgments into three dimensions: narrative–emotional congruence, technical integration, and thematic identity and originality. These dimensions are grounded in film music theory and enable fine-grained evaluation. For the dataset, we introduce VMAE-Sets (Video–Music Aesthetic Evaluation Datasets), derived from critically acclaimed films and their original soundtracks. To overcome the annotation bottleneck, we design a weakly supervised pipeline that leverages large language models to extract structured aesthetic evaluations from user reviews in movie databases. This pipeline generates multidimensional annotations for each video–music pair without requiring expert labeling.

The contributions of our work are follows:

1. We formulate video soundtrack aesthetic evaluation as a new task. This task aims to assess the artistic coordination between music and visual content. It provides a foundation for automatic quality assessment in film scoring and video production.
2. We propose MEMA, a multimodal model that captures audio–visual artistic relationships through crossmodal imagination. The model decomposes aesthetic judgments into three dimensions and achieves the strongest performance among all evaluated models, with average improvements of 18.137 percent in LCC and 17.866 percent in SRCC compared to the best-performing baseline.
3. We introduce VMAE-Sets, the first large-scale dataset for soundtrack aesthetic evaluation. A weakly supervised pipeline based on large language models enables scalable annotation without expert labeling.

## 2. Related Work

### 2.1. Audio Evaluation

Music quality evaluation has evolved from traditional acoustic assessment to modern data-driven approaches. Early work primarily focused on objective physical metrics such as audio fidelity and the signal-to-noise ratio [22,23]. These methods often relied on engineered features or signal-processing measures. With the advent of deep learning, researchers began to learn complex quality metrics directly from human subjective ratings [24]. Some studies employ the discriminator of a Generative Adversarial Network as a no-reference audio quality assessor [25]. This approach demonstrates strong alignment with human perception in certain settings. Other works have explored modeling higher-level musical attributes such as rhythm, harmony, and timbre [26,27,28]. Music emotion recognition has also been extensively studied [29]. However, most approaches remain single-modal. They analyze intrinsic musical qualities such as rhythmic stability but overlook the functional role of music within a visual narrative. This paradigm fails to capture the dynamic alignment between audio and visual elements like plot development or character emotions. Current metrics rely heavily on technical acoustic features rather than aesthetic dimensions like narrative congruence. Consequently, evaluating audio in isolation is insufficient. There is an urgent need for a crossmodal framework that effectively assesses the deep integration of audio and visual modalities.

### 2.2. Soundtrack Understanding

Currently, there are no specialized models designed specifically for soundtrack understanding. Researchers typically repurpose general video understanding models such as InternVideo, Video-LLaMA, and their successors [8,9,10,11,12,13,14,30,31]. These frameworks have achieved strong performance on semantic recognition tasks through large-scale multimodal pretraining. They are effective at capturing factual content such as object categories, actions, and events. However, they offer limited insight into aesthetic and emotional dimensions. These general foundation models fall short when applied to soundtrack evaluation. They lack the ability to model nuanced crossmodal relationships such as the narrative–emotional congruence between music and visual storytelling. Their training objectives are typically oriented toward recognition or captioning. This focus encourages semantic alignment but does not optimize for aesthetic qualities like emotional resonance, pacing, or thematic coherence in audio–visual compositions.

### 2.3. Video–Text and Video–Music Datasets

Alongside progress in video understanding, several video–music datasets have been proposed [32]. However, most were not designed with in-depth aesthetic evaluation in mind. SymMV [4] provides video–symbolic music pairs with detailed annotations of chords, melodies, and accompaniments. However, its scale is limited, and the music is largely restricted to piano renditions. This results in a lack of diversity in musical style and scene representation. The VidMuse V2M dataset [33] contains hundreds of thousands of video–music pairs covering movie trailers, advertisements, and documentaries. It is primarily used for video-to-music generation and matching. Its annotations focus on semantic-level alignment and do not provide explicit labels for aesthetic evaluation. HarmonySet [20] offers tens of thousands of samples annotated along dimensions including rhythm synchronization, emotional alignment, and thematic coherence. While it demonstrates more depth than earlier datasets, it primarily covers short-form videos rather than cinematic-length content. Although it includes labels such as emotional alignment, it does not comprehensively address more complex aesthetic dimensions of film soundtracks such as narrative–emotional congruence, technical integration, and thematic identity and originality.

### 2.4. Audio Aesthetic Evaluation Datasets

In the audio domain, several datasets have been proposed to quantify subjective evaluations of musical aesthetics. These datasets provide important references for our crossmodal setting. AES-Natural [26] unifies automatic quality assessment for speech, music, and sound. However, its clips are relatively short, and the evaluation dimensions focus on generic quality rather than detailed textual analysis of aesthetic properties. This makes it insufficient for studying complex musical structure in cinematic contexts. MusicEval [27] takes a step further by providing expert evaluations of AI-generated instrumental music. However, its evaluation dimensions remain relatively coarse and lack fine-grained granularity. Moreover, its text alignment metric becomes ineffective for assessing music that is not conditioned on textual prompts. This limits its applicability to video soundtrack evaluation. SongEval [28] introduces a multi-dimensional aesthetic evaluation of full songs with vocals. It proposes a five-dimensional framework annotated by professional musicians, including coherence, memorability, vocal naturalness, structural clarity, and overall musicality. While SongEval is an important milestone for single-modal song aesthetics, it is not designed for crossmodal tasks. All dimensions are evaluated in isolation on the audio itself without considering how the music aligns with visual content or supports narrative coherence. Thus, there remains a fundamental gap between existing audio-only aesthetic datasets and our goal of deeply fused video–soundtrack evaluation.

## 3. The VMAE-Sets Dataset

VMAE-Sets is a multimodal dataset comprising aligned text–audio–video samples for film soundtrack aesthetic evaluation. It assesses emotional and narrative congruence, technical integration, and thematic originality. Table 1 compares VMAE-Sets with representative datasets. Unlike SongEval [28] and HarmonySet [20], VMAE-Sets uniquely integrates video, OST, and commentary, providing a unified foundation for future research.

### 3.1. Data Collection

To ensure diversity and quality, we selected clips from critically acclaimed narrative films worldwide, covering a wide range of eras, genres, and production scales. For the audio component, we sourced official OST (original soundtrack) tracks from public distributions. User reviews of each soundtrack were collected from online platforms to serve as the raw textual material for later modeling and scoring. The final dataset contains 1170 soundtrack–video–text triplets, with a total duration of approximately 106.8 h, making it one of the largest datasets of its kind focused specifically on soundtrack aesthetics.

### 3.2. Video Processing

We adopted the Pretrained Conformers for Audio Fingerprinting method [35] to automatically match each OST to its corresponding appearance(s) in the film. After successful alignment, we extracted the matched video segments to ensure precise temporal synchronization between the music and the visuals. It is important to note that a single soundtrack track may be reused or segmented multiple times within a film, meaning that one OST track may correspond to 1 to *n* (where n≥2) video segments.

### 3.3. Definition of Evaluation Metrics

Inspired by prior work in film music theory and audio–visual studies [36,37,38], we define three evaluation metrics:

Narrative–Emotional Congruence (NEC) [36]: It measures the alignment between the emotional atmosphere created by the soundtrack and the narrative elements in the video, including plot progression, character psychology, and narrative tension. For example, cheerful music in a suspenseful or tragic scene would receive a lower NEC score.

Technical Integration (TI) [37]: It evaluates how well the soundtrack is integrated with other auditory components in the film, such as dialogue and sound effects, including aspects like mixing balance, masking, and clarity.

Thematic Identity and Originality (TIO) [38]: It focuses on the recognizability and creative uniqueness of the soundtrack’s musical theme—whether it possesses a distinct motive or leitmotif that connects with characters or plotlines and whether it avoids overly templated or formulaic orchestration.

### 3.4. Textual Review Processing and Scoring

To ensure objectivity, we processed user reviews using large language models (LLMs) weighted by “likes”. For each dimension, the LLMs generated (1) a score (1–10) for the given dimension, (2) a confidence score for each judgment, and (3) a corresponding textual explanation.

**Figure 1:**
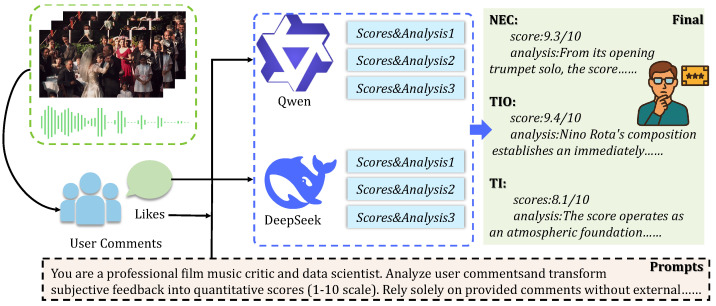
LLM-based scoring pipeline for VMAE-Sets. User comments and their “likes” are fed into two complementary LLMs (Qwen and DeepSeek), which generate multiple samples of scores and textual analyses for NEC, TI, and TIO. The confidence-weighted fusion produces final labels and rich explanations for each soundtrack.

We employed two LLMs (DeepSeek and Qwen) with three independent samplings per pair to reduce bias. Figure 1 illustrates the pipeline. We computed individual model scores using confidence-weighted averaging:

(1) $$s_{c}=\frac{\sum_{j}{\mathrm{score}}_{j}\times {\mathrm{conf}}_{j}}{\sum_{j}{\mathrm{conf}}_{j}},$$

where ${\mathrm{score}}_{j}$ and ${\mathrm{conf}}_{j}$ are the score and confidence of the *j*-th sample.

The final fused score from both LLMs was aggregated as

(2) $$s_{\mathrm{final}}=\frac{s_{\mathrm{model}1}\times {\mathrm{conf}}_{\mathrm{model}1}+s_{\mathrm{model}2}\times {\mathrm{conf}}_{\mathrm{model}2}}{{\mathrm{conf}}_{\mathrm{model}1}+{\mathrm{conf}}_{\mathrm{model}2}},$$

and the average confidence is defined as

(3) $${\mathrm{avg}}_{\mathrm{confidence}}=\frac{{\mathrm{conf}}_{\mathrm{model}1}+{\mathrm{conf}}_{\mathrm{model}2}}{2}.$$

The dataset covers 1170 samples across three dimensions. It contains over 6 million words of explanatory text. Sample lengths range from 70 to 1500 words, with an average of 900 words.

## 4. Model Construction

**Figure 2:**
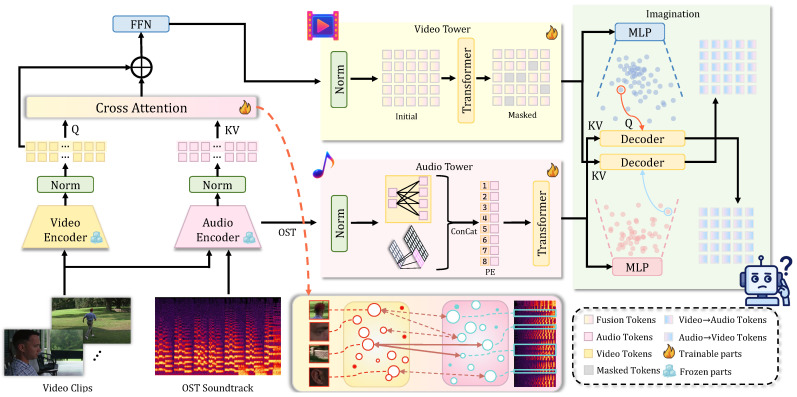
Crossmodal Imagination Module architecture. The CVAE-based module enables bidirectional reconstruction between audio and visual modalities through symmetric encoder–decoder paths, learning reversible semantic mappings in a unified latent space.

The MEMA model takes as input three types of data with the following correspondence: (1) The music sequence $A^{OST}=\left\{a_{1},\ldots ,a_{T}\right\}$ is derived from the pure OST—the isolated film soundtrack with non-musical elements (dialogue, ambient sounds, and sound effects) removed. (2) The *P* segments of ${\left\{V_{j}\right\}}_{j=1}^{P}$ are the visual content of the video, i.e., the image frames or visual features extracted from each video segment. (3) For each segment *j*, $A_{j}^{clip}$ denotes the audio track of the *j*-th video clip and the original mixed audio accompanying that clip, which includes dialogue, ambient sounds, and sound effects. Thus $A^{OST}$ and ${\left\{A_{j}^{clip}\right\}}_{j=1}^{P}$ provide two distinct audio views (isolated music vs. in-context mix), while ${\left\{V_{j}\right\}}_{j=1}^{P}$ provides the visual context; this design allows the model to assess how the pure soundtrack integrates with the environmental auditory elements in the final film. The model outputs three aesthetic scores (NEC, TI, and TIO) and textual explanations. The architecture consists of five main components: First, feature extraction modules encode audio and video inputs separately and fuse them at the segment level. Second, two encoding towers build global representations for music and scene collections. Third, a Crossmodal Imagination Module (Figure 2) establishes bidirectional semantic mappings between audio and visual modalities. Fourth, a Guided Cross-Attention Alignment Module enables music semantics to actively select relevant visual segments. Finally, prediction heads output aesthetic scores and generate textual explanations through a frozen language model. Training proceeds in two stages: self-supervised pretraining for crossmodal alignment and weakly supervised fine-tuning [39] for aesthetic prediction.

### 4.1. Feature Extraction and Local Fusion

**Audio Encoder:** Film music contains both abstract emotional semantics and concrete acoustic textures. Existing audio encoders typically capture only one aspect. We adopt a dual-pathway structure to model both. For semantic features, the OST $A^{OST}$ is sliced using a 10-s window with a 2-s stride to obtain *M* segments. Each segment is processed by CLAP [40] to extract $X_{clap}\in R^{M\times 512}$, then layer-normalized and projected:

(4) $$H_{clap}=W_{c}X_{clap}+b_{c}$$

where $W_{c}\in R^{512\times 768}$. For acoustic features, Librosa extracts 37-dimensional descriptors $X_{lib}\in R^{M\times 37}$ including MFCC, Chroma, RMS, zero-crossing rate, and timbral statistics. A three-layer 1D CNN processes these statistics into

(5) $$H_{motif}=f_{\mathrm{CNN}}\left(X_{lib}\right)\in R^{K\times 768}.$$

for each clip-level audio $A_{j}^{clip}$, features are concatenated and projected:

(6) $$x_{aud}^{\left(j\right)}=W_{a}\left[x_{\mathrm{clap}}^{\left(j\right)};x_{\mathrm{lib}}^{\left(j\right)}\right]+b_{a}$$

where $W_{a}\in R^{549\times 768}$.

**Video Encoder:** High-level semantic vectors alone make the model insensitive to visual aesthetics. We introduce a multi-layer mechanism to capture both global semantics and low-level properties. For each segment $V_{j}$, eight frames are sampled and input into InternVideo2 to obtain $x_{global}^{\left(j\right)}\in R^{768}$. The segment is also uniformly divided into five non-overlapping time intervals along the temporal axis to extract 40-dimensional low-level descriptors (eight dimensions per interval) including color, texture, and edge density, yielding $x_{low}^{\left(j\right)}\in R^{\begin{array}{c} 5\times 40 \end{array}}$. The features are concatenated:

(7) $$x_{vid}^{\left(j\right)}=\left[x_{global}^{\left(j\right)};\mathrm{Flatten}\left(x_{low}^{\left(j\right)}\right)\right]\in R^{968}.$$

**Segment-level Audio–Visual Fusion Module:** After obtaining features, segment-level fusion aligns the two modalities locally. Let $v_{i}=x_{vid}^{\left(i\right)}$ and $a_{i}=x_{aud}^{\left(i\right)}$. Linear transformations generate query, key, and value matrices, followed by cross-attention and residual fusion:

(8) $$\begin{array}{cc} Q & =W_{Q}v_{i},\;K=W_{K}a_{i},\;V=W_{V}a_{i},\\ \mathrm{Attention}(Q,K,V) & =\mathrm{Softmax}(QK^{⊤}/\sqrt{768})V,\\ H^{\prime } & =\mathrm{LayerNorm}(Q+\mathrm{Attention}(Q,K,V)),\\ H_{f} & =\mathrm{LayerNorm}(H^{\prime }+\mathrm{FFN}\left(H^{\prime }\right)). \end{array}$$

applying this to all *P* segments yields $H^{fusion}\in R^{P\times 768}$.

### 4.2. Audio Tower and Video Tower

**Audio Tower:** Local features must be aggregated into global representations. The Audio Tower integrates semantic and acoustic features to model the temporal progression of musical themes. The sequences $H_{clap}$ and $H_{motif}$ are concatenated and enhanced with the following sinusoidal positional encoding:

(9) $$H_{music}=\mathrm{PE}\left(\mathrm{Concat}\left(H_{clap},H_{motif}\right)\right)\in R^{(M+K)\times 768}.$$

a 6-layer Transformer [41] encoder with 12 heads and 3072-dimensional FFN outputs $H_{a}\in R^{(M+K)\times 768}$.

**Video Tower:** Film editing often does not follow a linear timeline due to flashbacks and intercuts. Strict temporal positional encoding may introduce noise. The Video Tower uses a masking mechanism instead of explicit positional encoding. The fusion features $H^{fusion}$ are layer-normalized. During encoding, a mask matrix $M_{scene}\in {\left\{0,1\right\}}^{P\times P}$ controls attention, ensuring narratively related segments attend to each other:

(10) $$\begin{array}{c} M_{scene}\left(i,j\right)=\left\{\begin{array}{cc} 0 & \mathrm{if}\;\mathrm{related},\\ -\infty & \mathrm{otherwise}, \end{array}\right.\\ \mathrm{Attention}\left(Q,K,V\right)=\mathrm{Softmax}(\left(QK^{⊤}+M_{scene}\right)/\sqrt{768})V. \end{array}$$

a 6-layer Transformer encoder outputs $H_{v}\in R^{P\times 768}$. This design allows narratively related segments to attend to each other regardless of their temporal positions.

### 4.3. Crossmodal Imagination Module

Existing multimodal methods align features through contrastive learning or concatenation. These approaches capture surface-level correlations but fail to model deep semantic connections. We propose a Crossmodal Variational Autoencoder (CVAE) that learns to generate one modality from another. This resembles human imagination and enables the model to understand how music evokes visual imagery. The module consists of two symmetric paths for bidirectional generation. The encoder maps input *H* (either $H_{a}$ or $H_{v}$) to latent parameters, and a Transformer decoder reconstructs features conditioned on the other modality:

(11) $$\begin{array}{c} {}[\mu ,\log\;\sigma^{2}]=f_{\mathrm{enc}}\left(H\right),\;z=\mu +\sigma ⊙\epsilon ,\;\epsilon ∼N\left(0,I\right),\\ {\hat{H}}_{v}=f_{\mathrm{dec}}\left(z|H_{a}\right)\in R^{P\times 768},\;{\hat{H}}_{a}=f_{\mathrm{dec}}\left(z|H_{v}\right)\in R^{(M+K)\times 768}. \end{array}$$

the loss function combines reconstruction and KL divergence, with

(12) $$L_{\mathrm{CVAE}}=∥H_{v}-{\hat{H}}_{v}{∥}_{2}^{2}+{∥H_{a}-{\hat{H}}_{a}∥}_{2}^{2}+\lambda_{KL}D_{KL}\left(q\left(z|H\right)∥N\left(0,I\right)\right).$$

Through bidirectional reconstruction, the model learns a unified latent space where semantically related features are mapped to nearby regions. The CVAE uses a symmetric encoder–decoder architecture: the encoder is a 3-layer Transformer (six heads; 2048-d feed-forward) that maps $H_{a}$ or $H_{v}$ to latent parameters; the decoder mirrors it with a 3-layer Transformer that performs cross-attention between the latent sample *z* and the conditioning modality. The latent dimension is set to $z\in R^{256}$ to capture high-level semantic correspondences while avoiding overfitting to low-level details. The symmetric encoder–decoder structure ensures that both bidirectional pathways have equivalent capacity.

### 4.4. Guided Cross-Attention Alignment Module

**Figure 3:**
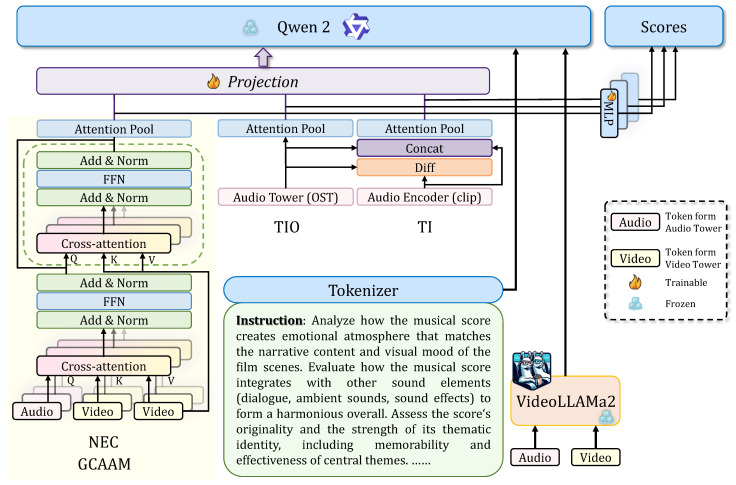
Processed tokens from the Audio and Video Towers are fed into the GCAAM. Combined with VideoLLaMA2 vectors, the model generates the final scores and text.

Segment-level fusion captures local correspondence. However, film scores exhibit long-range thematic structures spanning multiple scenes. A musical theme may relate to temporally distant scenes. Local fusion cannot capture this global narrative role. We introduce the Guided Cross-Attention Alignment Module (GCAAM) (Figure 3), where music semantics guide the attention process by serving as queries to selectively attend to relevant visual segments. This “guidance” is realized through two key design choices that distinguish the GCAAM from standard cross-attention: (1) We use only the first *M semantic* tokens ($H_{a}^{\left(M\right)}$) from the Audio Tower. These tokens are derived from CLAP embeddings and were aligned through CVAE pretraining to capture global musical structure and thematic progression. This selective use of semantically rich tokens, rather than all audio features, enables the model to focus on musically meaningful patterns. (2) The audio-to-video attention is asymmetric: music acts as the sole query source, while video serves as the key and value, establishing a music-driven selection mechanism rather than bidirectional mutual attention. We extract the first *M* semantic tokens from $H_{a}$, denoted $H_{a}^{\left(M\right)}\in R^{M\times 768}$, as queries. The scene representation $H_{v}$ serves as keys and values, and cross-attention is computed as follows:

(13) $$\begin{array}{cc} Q_{m} & =W_{Q}H_{a}^{\left(M\right)},\;K_{v}=W_{K}H_{v},\;V_{v}=W_{V}H_{v},\\ H^{\prime } & =\mathrm{Softmax}\left(Q_{m}K_{v}^{⊤}/\sqrt{768}\right)V_{v}. \end{array}$$

The output passes through residual connections, layer normalization, and FFN to produce $H_{aligned}\in R^{M\times 768}$. This mechanism establishes a global mapping between musical themes and scene contexts. The attention weights can be visualized to analyze which scenes each musical segment attends to.

### 4.5. Prediction Heads

**Score Prediction Heads:** The model outputs three aesthetic scores from features that best reflect each dimension. NEC measures narrative–emotional congruence and is computed from

(14) $$u_{NEC}={\mathrm{Pool}}_{t}\left(H_{aligned}\right)\in R^{768}.$$

TIO evaluates thematic identity and originality from

(15) $$u_{TIO}={\mathrm{Pool}}_{t}\left(H_{a}\right)\in R^{768}.$$

TI assesses technical integration by comparing global and local audio, with

(16) $$u_{a}^{OST}={\mathrm{Pool}}_{t}\left(H_{a}\right),u_{a}^{clip}={\mathrm{Pool}}_{j}\left(\left\{x_{aud}^{\left(j\right)}\right\}\right),\Delta =u_{a}^{OST}-u_{a}^{clip},\;\mathrm{and}\;u_{TI}=\left[u_{a}^{OST};u_{a}^{clip};\Delta \right]\in R^{2304}.$$

each metric uses a two-layer MLP ($d_{in}\to 512$ with ReLU and Dropout 0.1, then 512→1 with Sigmoid). Training uses confidence-weighted MSE, where

(17) $$L_{score}^{t}=\frac{1}{N}\sum_{i}w_{i}^{t}{\left(y_{i}^{t}-{\hat{y}}_{i}^{t}\right)}^{2}.$$

**LLM Output Head:** The model also generates textual explanations. Separate MLP projectors map task vectors to the language embedding space as follows:

(18) $$\phi_{NEC}:R^{768}\to R^{1536}\to R^{3584},\phi_{TIO}:R^{768}\to R^{1536}\to R^{3584},\phi_{TI}:R^{2304}\to R^{1536}\to R^{3584}.$$

projected embeddings are concatenated with VideoLLaMA2 features and input to a frozen Qwen2-7B [42] dataset with task-specific prompts. Only projectors are trainable. Cross-entropy loss is calculated as

(19) $$L_{text}=-\sum_{t}y_{t}\log p\left(y_{t}|y_{<t},H_{task}\right).$$

### 4.6. Training Strategy

**Self-supervised Pretraining:** The first stage performs crossmodal alignment without labels. The loss function combines reconstruction, KL divergence, and alignment regularization to yield

(20) $$\begin{array}{cc} L_{pre} & =\lambda_{rec}L_{rec}+\lambda_{KL}L_{KL}+\lambda_{align}L_{align},\\ \mathrm{where}\;L_{rec} & ={∥H_{v}-{\hat{H}}_{v}∥}_{2}^{2}+{∥H_{a}-{\hat{H}}_{a}∥}_{2}^{2},\\ L_{KL} & =D_{KL}\left(q\left(z|H\right)∥N\left(0,I\right)\right),\\ \mathrm{and}\;L_{align} & =\mathrm{MSE}(A_{attn},{\bar{A}}_{attn}) \end{array}$$

where $A_{attn}$ denotes GCAAM attention weights and ${\bar{A}}_{attn}$ is their segment-wise average. To prevent posterior collapse during CVAE training, we employ a KL annealing strategy for $\lambda_{KL}$. Specifically, $\lambda_{KL}$ linearly increases from zero to a maximum value $\lambda_{KL}^{max}$ over the first $N_{warmup}$ epochs: $\lambda_{KL}^{\left(t\right)}=\min\left(t/N_{warmup},1\right)\cdot \lambda_{KL}^{max}$. This allows the model to first learn effective reconstruction before gradually introducing regularization. The final target value $\lambda_{KL}^{max}=0.01$ was selected via grid search over {0.001,0.01,0.05,0.1,0.5} on the validation set, balancing reconstruction quality and latent space smoothness. The settings are as follows: $N_{warmup}=10$ epochs, $\lambda_{rec}=1.0$, and $\lambda_{align}=0.1$.

**Weakly Supervised Fine-tuning:** The second stage adapts the model to aesthetic prediction. CVAE parameters are frozen. The loss function combines score regression and text generation using

(21) $$L_{score}=\sum_{t}\omega_{t}\cdot \mathrm{MSE}\left(s_{t},{\hat{s}}_{t}\right)\;\mathrm{and}\;L_{text}=\sum_{t}\gamma_{t}\cdot \mathrm{CE}\left(y_{t},{\hat{y}}_{t}\right)$$

where t∈{NEC,TIO,TI}. Correlation regularization stabilizes training:

(22) $$L_{fine}=\alpha_{score}L_{score}+\beta_{text}L_{text}+\lambda_{lcc}\left(1-\rho \right)$$

where ρ represents Pearson’s correlation. Training uses AdamW with a learning rate of $5\times 10^{-5}$ and mixed precision.

## 5. Experiments and Analysis

### 5.1. Experimental Setup

To validate the effectiveness of MEMA, we conducted experiments on the VMAE-Sets dataset. Since no existing models are tailored for soundtrack aesthetic evaluation, we compare MEMA against several categories of baselines: (1) the non-reference audio quality model (MOSNet); (2) two multimodal video quality models (FastVQA and PTM-VQA); (3) the general-purpose multimodal alignment model (ImageBind), which learns a joint embedding space across six modalities including audio and video; and (4) the video–music matching model (VidMuse), a state-of-the-art approach for video-to-music generation and retrieval tasks. MOSNet is only evaluated on TI, as NEC and TIO require video processing. FastVQA and PTM-VQA were originally designed for single-score assessments, while ImageBind and VidMuse were designed for crossmodal alignment rather than aesthetic scoring. We adapt all multimodal models by adding three independent score heads while preserving their original feature extraction structures. The evaluation metrics include the Linear Correlation Coefficient (LCC), Spearman’s Rank Correlation Coefficient (SRCC), Kendall’s Rank Correlation Coefficient (KTAU), the Mean Squared Error (MSE), and the Mean Absolute Error (MAE). These metrics measure the correlation and error between predicted scores and human annotations. The training batch size is 32. Results are shown in Table 2.

### 5.2. Overall Performance Comparison

The MEMA model significantly outperforms all baseline methods across all three aesthetic dimensions. Compared to VidMuse, the strongest baseline, MEMA achieves substantial improvements: in the NEC dimension, LCC improves by 24.528%, SRCC by 22.760%, and KTAU by 30.824%. In the TI dimension, LCC improves by 17.763%, SRCC by 16.553%, and KTAU by 6.510%. Based on TIO, the improvements are 12.121% for LCC, 14.286% for SRCC, and 9.337% for KTAU. Notably, ImageBind and VidMuse outperform video quality assessment models (FastVQA and PTM-VQA) on correlation metrics, demonstrating that crossmodal alignment capabilities are beneficial for this task. However, these general-purpose multimodal models still fall short of MEMA, as they lack task-specific mechanisms for aesthetic evaluation. VidMuse shows stronger performance than ImageBind, likely due to its specialization in video–music correspondence learning. Nevertheless, MEMA surpasses VidMuse by a significant margin, validating the effectiveness of our CVAE-based crossmodal imagination module and guided cross-attention alignment mechanism. MEMA shows particularly strong performance in the NEC and TI dimensions, highlighting its advantages in crossmodal narrative consistency and audio–visual fusion quality. Performance in TIO is relatively lower across all models. We attribute this to the nature of thematic originality, which depends heavily on internal musical structure and artistic style. These aspects are more subjective and harder to annotate reliably, resulting in higher variance in the dataset. Nevertheless, all three dimensions maintain relatively high correlation metrics. This suggests that MEMA captures the holistic aesthetic features of film music from multiple perspectives.

### 5.3. Ablation

To evaluate the contribution of each key module to the overall performance, we conduct a systematic ablation study on VMAE-Sets. Using the MEMA model as the baseline, we remove or replace individual core components to examine their effects on the aesthetic evaluation tasks. The ablation settings are as follows:

(1) w/o CVAE: The Crossmodal Imagination Module is removed, and the model performs crossmodal mapping using feature concatenation only. (2) w/o GCAAM: The Guided Cross-Attention Alignment Module is replaced with a standard attention mechanism. (3) w/o Acoustic Branch: The acoustic feature branch is removed, retaining only the semantic audio encoding pathway. (4) w/o AV-Fusion: The segment-level audio–visual fusion layer is removed, and the model performs global-level alignment only. (5) Shared Head: All three dimensions share a single scoring head rather than using independent branches.

All experiments are conducted using the same data split and training procedure. LCC and SRCC are used as the evaluation metrics.

As shown in Table 3, the results show that CVAE has the most significant impact. Removing it leads to SRCC drops of 46.131%, 33.675%, and 43.295% in the NEC, TI, and TIO dimensions respectively, highlighting its central role in establishing deep semantic mappings between music and visual content. Interestingly, the GCAAM primarily affects the NEC dimension (25.109% drop) while having minimal impact on TI (0.146%) and TIO (0.383%), suggesting its specialized role in narrative–emotional alignment. Eliminating the Acoustic Branch results in consistent drops of 21.022%, 26.647%, and 24.138%, reflecting its complementary contribution to audio–visual integration quality. Removing AV-Fusion causes substantial degradation across all dimensions, with SRCC decreasing by 32.409%, 36.310%, and 25.670%, respectively. The Shared Head configuration has the smallest effect, causing only 4.526%, 3.075%, and 2.490% degradation, indicating that the three aesthetic dimensions share certain feature patterns.

### 5.4. Robustness Analysis

**Figure 4:**
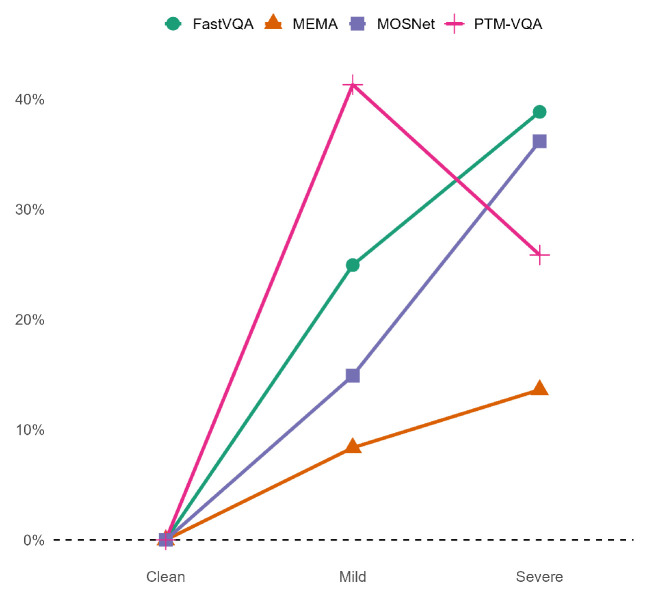
Use Avg LCC as the evaluation metric.

To evaluate the reliability and generalization ability of MEMA in practical scenarios, we assess performance variations under different video and audio quality conditions. All models are trained using the same dataset and settings. During testing, input video or audio signals are subjected to degradation. Video degradation is achieved by downscaling the resolution from the original 480p to 360p (mild) and 240p (severe). Audio degradation is implemented by reducing the bitrate to 128 kbps, 64 kbps, and 32 kbps. These constitute three quality levels: normal, mildly degraded, and severely degraded. As shown in Figure 4, MEMA consistently exhibits the lowest degradation rate under all quality levels. Its performance degradation curve remains the most stable. Compared to baseline models, MEMA demonstrates smaller performance drops in response to reduced input quality. This indicates that MEMA maintains higher prediction stability even under low-quality conditions. This robustness makes MEMA more suitable for real-world applications such as soundtrack evaluation and retrieval, where input quality may fluctuate significantly.

### 5.5. Visualization Analysis

**Figure 5:**
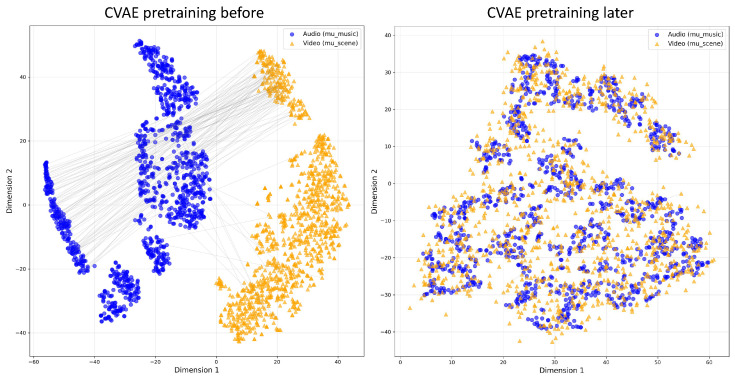
Distribution of audio and visual samples in the latent space before and after CVAE pretraining.

To explore the internal working mechanism of MEMA, we visualize its latent space to examine how music and video modalities are aligned. Figure 5 compares the distribution of audio and visual samples before and after CVAE pretraining. Blue circles represent music segments. Orange triangles represent video segments, and gray lines connect semantically matched pairs. Before pretraining, audio and video samples form separate clusters with minimal overlap. The long gray lines indicate that semantically related samples are distant in the latent space due to a lack of alignment. After CVAE pretraining, paired audio and video embeddings are located much closer to each other. The gray lines are significantly shortened. This demonstrates that the model has learned to map semantically related music and video into neighboring regions. The result confirms that CVAE pretraining effectively brings different modalities into alignment. This alignment facilitates more efficient attention allocation by the downstream GCAAM.

**Figure 6:**
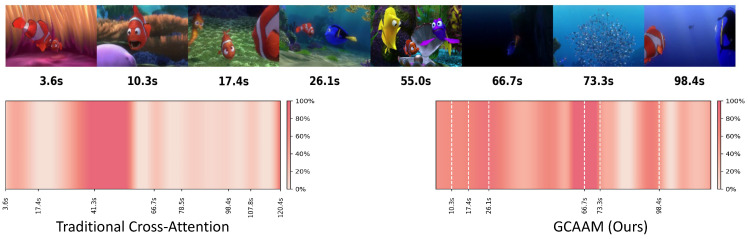
Attention patterns of the GCAAM and traditional cross-attention.

To validate the effectiveness of the GCAAM, we compare its attention patterns with standard cross-attention. In Figure 6 (left side), traditional attention weights are scattered and easily influenced by low-level features such as motion or color. This makes it difficult to focus on narrative-critical moments in the video. The model fails to establish a strong link between music emotion and narrative progression. In contrast, the GCAAM introduces global semantics from the entire soundtrack as guidance. This results in a distinctly different attention pattern. High-weight regions concentrate around key narrative events: 10.3 s for main character appearance, 26.1 s for parent–child interaction, and 66.7 s for crisis exploration. These regions correspond closely with emotional shifts in the music. They form continuous bands of attention over time. The model consistently enhances attention in segments with recurring narrative motifs. This showcases its understanding of long-term musical structure.

### 5.6. Pairwise Matching Discrimination

**Figure 7:**
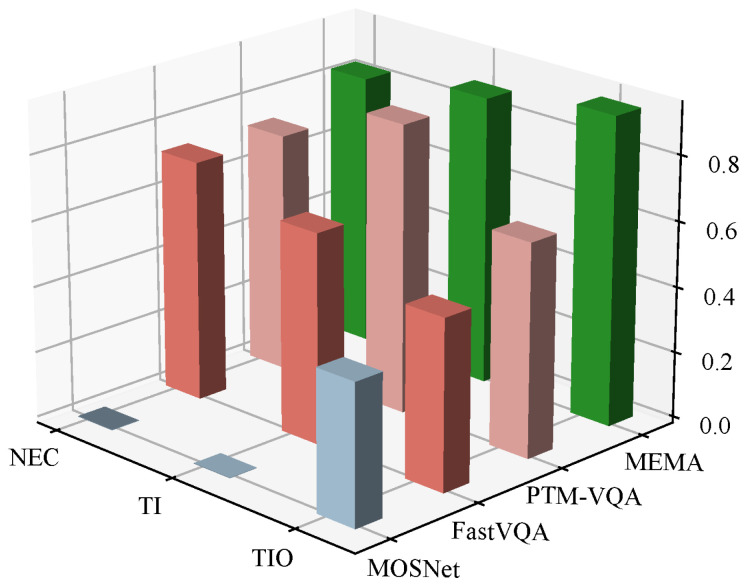
Pairwise matching accuracy for each dimension.

We compute the pairwise matching accuracy based on the three scoring heads to evaluate discriminative capability across aesthetic dimensions. For each video segment $V_{i}$, we construct a ground-truth pair $\left(V_{i},M_{i}\right)$ and a randomly mismatched pair $\left(V_{i},M_{j}\right)$, where j≠i. From the scoring head for dimension d∈{NEC,TI,TIO}, we obtain two scores: $s_{i,d}^{\mathrm{match}}$ and $s_{i,d}^{\mathrm{mismatch}}$. If $s_{i,d}^{\mathrm{match}}>s_{i,d}^{\mathrm{mismatch}}$, we count this as a correct prediction. The pairwise matching accuracy is the ratio of correct predictions over all test samples. Since MOSNet only outputs scores related to music themes, it is included solely in the TIO comparison. NEC and TI are evaluated using FastVQA, PTM-VQA, and MEMA. As shown in Figure 7, MEMA achieves superior pairwise matching accuracy across all three dimensions compared to baseline methods.

### 5.7. Crossmodal Retrieval on SymMV

To isolate and evaluate the effectiveness of the proposed Crossmodal Variational Autoencoder (CVAE), we perform a video-to-music retrieval task on the SymMV dataset. Since SymMV contains paired video and background music tracks without aesthetic scores, it is highly suitable for assessing crossmodal semantic alignment.

Experimental Setup: During training, the CVAE is optimized using the matched video–audio pairs from the SymMV training set to minimize reconstruction loss and KL divergence. During testing, for each query video, we utilize the CVAE to map its visual representation to the latent space *z* and decode it to generate an “imagined” audio feature ${\hat{H}}_{a}$. We then compute the cosine similarity between ${\hat{H}}_{a}$ and the real audio features $H_{a}$ of all music tracks in the test set database. The performance is measured by Recall at K (R@1, R@5, and R@10) and the Median Rank (MR).

Results: We compare our CVAE with a standard dual-encoder model optimized via InfoNCE loss (contrastive learning) and the strong multimodal foundation model, ImageBind. As shown in Table 4, MEMA-CVAE achieves the highest precision in top-ranked results (an R@1 of 19.5% and an R@5 of 41.8%) and the best Median Rank. Interestingly, while ImageBind performs exceptionally well in broad retrieval (an R@10 of 54.3%), due to its massive pretraining corpus, our CVAE yields more accurate exact matches. This demonstrates that generating an “imagined” feature conditioned on the visual input provides a tighter semantic bound than simply learning a joint embedding space.

### 5.8. Inference Efficiency Analysis

To verify that MEMA maintains high inference efficiency while ensuring performance, we analyze the runtime of key components. All experiments are conducted on an RTX 4060 platform. As shown in Table 5, MEMA achieves an average inference time of approximately 2.49 milliseconds per sample. This corresponds to a throughput of 401 frames per second, which is well above the threshold for real-time processing. The majority of computation time is spent on feature extraction. The Audio Tower and Video Tower account for approximately 1.27 ms and 0.67 ms respectively. The Clip Binder module consumes only 0.24 ms due to its lightweight fusion strategy. The GCAAM requires just 0.16 ms to compute. The final aggregation and scoring prediction modules together take less than 0.16 ms. Both Attention Pooling and scoring heads operate in the sub-millisecond range. This indicates that the decision-making phase introduces negligible additional latency. These results demonstrate that MEMA offers a favorable balance between prediction quality and computational cost. This makes it suitable for time-sensitive applications such as real-time soundtrack evaluation and retrieval systems.

### 5.9. Human Evaluation vs. Model Prediction

**Figure 8:**
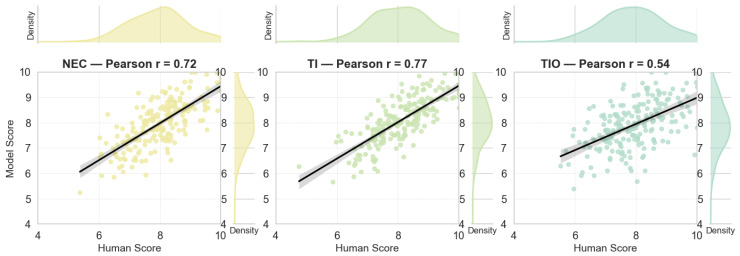
Scatter plots of “Human Score vs. Model Prediction” across the three dimensions.

To evaluate the reliability of MEMA in real-world perceptual tasks, we conducted a human evaluation involving 20 volunteers with backgrounds in music and film. The volunteers rated 200 video–soundtrack pairs across three dimensions on a scale of 0 to 10. We recorded MEMA predictions for the same samples and performed consistency analysis. As shown in Figure 8, scatter plots across three dimensions show points clustered near the diagonal line, indicating a clear linear relationship. The Pearson correlation is highest on TI at 0.77, suggesting good alignment with human judgment in perceiving technical integration of dialogue, sound effects, and music. NEC shows a correlation of 0.72, indicating strong performance in capturing the emotional atmosphere and narrative alignment. TIO yields a lower correlation of 0.54 but remains statistically significant. This implies moderate ability to identify musical thematicity and originality. This dimension tends to be more dependent on subjective aesthetics and exhibits greater individual variation. The scatter density maps show that both human scores and model predictions concentrate in the 6 to 9 range, forming a slightly right-skewed distribution. This indicates high overall sample quality and close alignment between model and human judgment on high-scoring samples. MEMA demonstrates human-aligned perceptual results across multiple subjective dimensions with stable scoring behavior.

### 5.10. LLM Training Effectiveness

**Figure 9:**
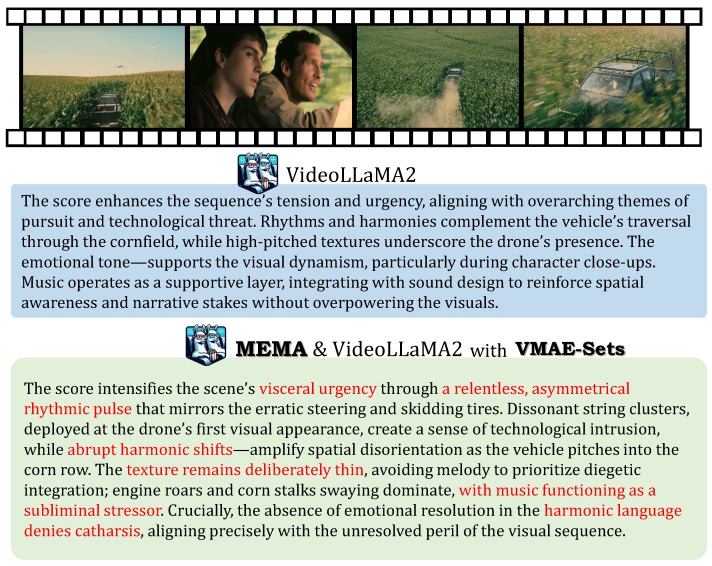
Comparison of VideoLLaMA2’s original and fine-tuned performance.

We adopt VideoLLaMA2 as the baseline model for large language model evaluation. The original video–audio–text generation flow of VideoLLaMA2 is kept frozen during training. Using VMAE-Sets as the training material, we train only the projection heads from the NEC, TI, and TIO vectors to the Qwen2 model space. The comparison results are shown in Figure 9. In its original form, VideoLLaMA2 struggles to capture artistic relationships between soundtrack and the emotional–narrative structure. This limitation may be due to lack of understanding or representation of multimodal aesthetic semantics. With VMAE-Sets and carefully extracted task-specific features, the model generates more detailed and context-aware evaluations. The fine-tuned output demonstrates improved ability to articulate alignment between soundtrack, narrative, and emotional cues. These enriched assessments provide valuable insights for future soundtrack composition. They potentially support more intelligent and perceptually guided music scoring in multimedia applications.

## 6. Conclusions

This work addresses the challenge of evaluating the aesthetic quality of film soundtracks by introducing a multidimensional assessment framework that accounts for both narrative and technical coherence between music and video. We propose MEMA, a multimodal model that rethinks soundtrack evaluation as a crossmodal alignment problem, guided by long-range musical semantics and clip-level audio–visual interactions.

Our experimental results demonstrate that MEMA outperforms state-of-the-art video and audio quality assessment models across all three proposed dimensions—narrative–emotional congruence (NEC), technical integration (TI), and thematic identity and originality (TIO). Through ablation studies, we validated the critical roles of the CVAE imagination module and guided attention alignment (GCAAM). The model exhibits strong robustness under varying input quality, closely aligns with human judgment in subjective evaluation, and generates semantically rich assessments via a Qwen2-enhanced LLM head.

These findings suggest that multimodal aesthetic evaluation benefits from modeling not only fused sensory signals but also their structured narrative alignment. MEMA sets a foundation for intelligent soundtrack assessment and recommendation and offers new perspectives for future research in computational aesthetics, crossmodal generation, and music-driven video understanding.

## Figures and Tables

**Table 1:** Comparison of representative datasets for music evaluation.

Dataset	Total Hours	Video	Pure Audio ^1^	Text ^2^	Evaluation Score	Task Focus
SymMV [4]	76.5	✓	✓	×	×	Video background-music generation
MusicEval [27]	16.67	×	✓	×	✓	Text-to-music evaluation
SongEval [28]	140.32	×	✓	×	✓	Music aesthetic evaluation
MERP [34]	—	×	✓	×	✓	Songs with valence ratings
AES-Natural [26]	∼500	×	✓	×	✓	Audio aesthetic evaluation
HarmonySet [20]	458.8	✓	×	✓	×	Narrative and thematic alignment
VMAE-Sets (ours)	106.8	✓	✓	✓	✓	Soundtrack aesthetic evaluation

Notes: ^1^ “Pure Audio” refers to the isolated soundtrack with non-musical elements removed for video-based datasets; for audio-only datasets, it refers to the original audio. ^2^ “Text” refers to aesthetic or quality assessment texts accompanying a music segment.

**Table 2:** Comparison of Models on Three Aesthetic Dimensions.

Model	Dimension	LCC ↑	SRCC ↑	KTAU ↑	MSE ↓	MAE ↓
MOSNet	TI	0.235	0.356	0.286	0.988	1.132
	NEC	0.318	0.317	0.286	1.263	1.135
FastVQA	TIO	0.324	0.452	0.307	0.683	**0.637**
	TI	0.437	0.405	0.274	0.578	0.716
	NEC	0.213	0.198	0.217	0.432	0.579
PTM-VQA	TIO	0.426	0.384	0.269	0.675	0.762
	TI	0.332	0.578	0.408	0.473	0.681
	NEC	0.518	0.493	0.382	0.618	0.702
ImageBind	TIO	0.463	0.441	0.308	0.628	0.719
	TI	0.542	0.508	0.355	0.538	0.701
	NEC	0.583	0.558	0.425	0.545	0.632
VidMuse	TIO	0.495	0.483	0.332	0.601	0.638
	TI	0.608	0.586	0.384	0.501	0.695
	NEC	**0.726**	**0.685**	**0.556**	**0.458**	**0.537**
**MEMA (ours)**	TIO	**0.555**	**0.552**	**0.363**	**0.568**	0.761
	TI	**0.716**	**0.683**	**0.409**	**0.472**	**0.681**

↑: higher is better; ↓: lower is better. Bold: best value in that column and dimension (e.g., highest SRCC in NEC).

**Table 3:** Ablation Study Results.

Model Variant	SRCC			LCC		
	**NEC**	**TI**	**TIO**	**NEC**	**TI**	**TIO**
MEMA-full	0.685	0.683	0.522	0.726	0.716	0.555
w/o CVAE	0.369	0.453	0.296	0.424	0.472	0.322
w/o GCAAM	0.513	0.682	0.520	0.556	0.710	0.550
w/o Acoustic Branch	0.541	0.501	0.396	0.583	0.512	0.423
w/o AV-Fusion	0.463	0.435	0.388	0.495	0.458	0.416
Shared Head	0.654	0.662	0.509	0.702	0.698	0.537

**Table 4:** Comparison of Cross-modal Video-Music Retrieval Performance on the SymMV Dataset. Higher Recall (R@K) and lower Median Rank (MR) indicate better performance.

Model	R@1 ↑	R@5 ↑	R@10 ↑	MR ↓
Dual-Encoder (Contrastive)	13.2%	32.5%	48.7%	12.0
ImageBind	17.6%	38.2%	**54.3%**	9.0
**MEMA-CVAE (ours)**	**19.5%**	**41.8%**	53.6%	**7.0**

↑: higher is better; ↓: lower is better. Bold: best value in that column.

**Table 5:** Inference time breakdown of MEMA.

Main Steps	FPS	Milliseconds
Clip Binder		0.23567 ms
Music Tower		1.27006 ms
Scene Tower		0.66514 ms
GCAAM		0.16491 ms
Aggregation & Prediction		0.15790 ms
– Temporal attention pooling		0.07760 ms
– Score prediction head		0.08029 ms
Total Inference Time per sample	401.0	2.49368 ms

## Data Availability

The raw data supporting the conclusions of this article will be made available by the authors upon request.

## Abbreviations

The following abbreviations are used in this manuscript:

NEC	Narrative–Emotional Congruence
TI	Technical Integration
TIO	Thematic Identity and Originality
MEMA	Multimodal Aesthetic Evaluation of Music
VMAE-Sets	Video–Music Aesthetic Evaluation Datasets
OST	Original Soundtrack
CVAE	Conditional Variational Autoencoder
GCAAM	Guided Cross-Attention Alignment Module
LLM	Large Language Model
LCC	Linear Correlation Coefficient
SRCC	Spearman’s Rank Correlation Coefficient
KTAU	Kendall Rank Correlation Coefficient
MSE	Mean Squared Error
MAE	Mean Absolute Error
MFCC	Mel-frequency Cepstral Coefficients
RMS	Root Mean Square
CNN	Convolutional Neural Network
MLP	Multi-Layer Perceptron
FFN	Feed-Forward Network
PE	Positional Encoding
